# Effects of *δ*‐aminolevulinic acid dehydratase silencing on the primary and secondary metabolisms of citrus

**DOI:** 10.1002/pld3.72

**Published:** 2018-07-16

**Authors:** Nabil Killiny, Faraj Hijaz, Yasser Nehela, Subhas Hajeri, Siddarame Gowda

**Affiliations:** ^1^ Department of Plant Pathology Citrus Research and Education Center IFAS University of Florida Lake Alfred Florida; ^2^ Present address: Citrus Pest Detection Program Central California Tristeza Eradication Agency Tulare California

**Keywords:** *δ*‐aminolevulinic acid dehydratase, abscisic acid, citrus, leaf pigments, virus‐induced gene silencing

## Abstract

*δ*‐aminolevulinic acid dehydratase (ALAD) is an important enzyme in tetrapyrrole synthesis. ALAD combines two *δ*‐aminolevulinic acid (*δ*‐ALA) molecules to form the pyrrole molecule, porphobilinogen, an important precursor for plant pigments involved in photosynthesis, respiration, and nutrient uptake. In this study, we investigated the effects of silencing of *ALAD* gene on citrus leaf pigments and metabolites. The ALAD enzyme was inhibited using virus‐induced gene silencing (VIGS) technology using *citrus tristeza* virus (CTV). *δ*‐ALA accumulated in citrus plants inoculated with the recombinant virus (CTV‐tALAD) to silence *ALAD* and resulted in discrete yellow spots (yellow islands) and necrosis in leaves and stems. The levels of chlorophylls, starch, sucrose, *trans*‐ and *cis*‐violaxanthin, and *α*‐ and *β*‐cryptoxanthin were reduced in CTV‐tALAD plants, whereas zeaxanthin was increased. The increase in zeaxanthin and the decrease in its precursors indicated that the reduction in chlorophylls resulted in light damage. Salicylic acid and jasmonic acid levels, as well as emission of (*E*)‐*α*‐bergamotene and (*E*)‐*β*‐farnesene, increased in CTV‐tALAD plants indicating these plants were under stress. Our results showed that silencing of *ALAD* induces stress in plants and that VIGS using mild CTV strains is a promising technique to study biological function of citrus genes.

## 1 INTRODUCTION

Tetrapyrroles are macrocyclic compounds that contain four pyrrole rings with various structural alternatives and numerous biological functions in prokaryotes and eukaryotes. Higher plants possess four classes of tetrapyrroles that play important roles in many biological processes; chlorophyll is essential for photosynthesis, heme is necessary for respiration, siroheme is necessary for sulfur and nitrogen assimilation, and phytochromobilin is required for light sensing (Tanaka & Tanaka, 2007). Tetrapyrrole biosynthesis is a multistep and a multibranched pathway; the first six steps (from glutamate to uroporphyrinogen III) are required by all classes of tetrapyrroles, whereas the next three steps (from uroporphyrinogen III to protoporphyrin IX) are common to phytochromobilin, heme, and chlorophyll biosynthesis (Tanaka & Tanaka, 2007).

In the first step of tetrapyrrole biosynthesis, glutamyl‐tRNA synthetase attaches glutamate to t‐RNA to form tRNAGlu complex. In the second step, the carboxyl group of glutamyl‐tRNA is converted to a formyl group by glutamyl‐tRNA reductase to generate glutamate‐1‐semialdehyde (GSA). In the third step, GSA is converted into *δ*‐ALA by GSA aminotransferase (GSA‐AT). In the fourth step, two *δ*‐ALA molecules are combined together by ALA‐dehydratase (ALAD) to form a pyrrole molecule, porphobilinogen (PBG) (Tanaka & Tanaka, 2007). The *δ*‐ALA does not accumulate in developing leaves and is directly converted to PBG by ALAD. However, the addition of levulinic acid, a specific competitive inhibitor of ALAD, can induce the accumulation of *δ*‐ALA in greening tissues (Kumar & Söll, 2000).

Functional genomics using mutant plants have been extensively used to investigate the regulatory mechanism of tetrapyrrole synthesis and the role of various enzymes in this pathway. Most of these studies were performed on *Arabidopsis thaliana* (Espineda, Linford, Devine, & Brusslan, 1999; Frick, Su, Apel, & Armstrong, 2003; Hirono & Rédei, 1963; Ishikawa, Okamoto, Iwasaki, & Asahi, 2001; Kumar & Söll, 2000; Molina et al., 1999; Nagata, Tanaka, Satoh, & Tanaka, 2005; Tanaka & Tanaka, 2005) and *Nicotiana tabacum* (Alawady & Grimm, 2005; Höfgen et al., 1994; Kruse, Mock, & Grimm, 1995; Mock, Keetman, Kruse, Rank, & Grimm, 1998; Mock et al., 1999; Papenbrock, Mock, Tanaka, Kruse, & Grimm, 2000; Papenbrock et al., 2001). Other plants species such as *Zea mays* (Hu, Yalpani, Briggs, & Johal, 1998), *Hordeum vulgare* (Jensen et al., 1996; Rzeznicka et al., 2005), *Pisum sativum* (Weller, Terry, Rameau, Reid, & Kendrick, 1996; Weller, Terry, Reid, & Kendrick, 1997), and *Brassica napus* (Tsang et al., 2003) were also investigated. In general, inhibition of *δ*‐ALA formation in mutant plants reduced chlorophyll and heme formation, but did not result in necrotic lesions (Tanaka & Tanaka, 2007). On the other hand, inhibition of later steps in the tetrapyrrole pathway (uroporphyrinogen III decarboxylase to protoporphyrinogen IX oxidase) increased the level of intermediate molecules and resulted in necrotic lesions upon illumination (Tanaka & Tanaka, 2007).

Although the first three enzymes that lead to *δ*‐ALA formation have been extensively targeted (Kim et al., 2005; Kumar & Söll, 2000; Tsang et al., 2003), ALAD rarely has been studied in plants (Chai et al., 2017). In humans, ALAD is necessary for heme biosynthesis and its activity can be inhibited by environmental toxins such as lead (Scinicariello et al., 2007). Lead deactivates ALAD by displacing the zinc from the active site of the enzyme and consequently leads to the accumulation of *δ*‐ALA, which can act as a *γ*‐aminobutyric acid (GABA) receptor agonist in the nervous system resulting in neuropathogenic effects (Brennan & Cantrill, 1979). ALAD enzyme has been isolated and characterized in several organisms, including human, mouse, yeast, bacteria, and many plant species such as pea, soybean, and spinach (Kaczor, Smith, Sangwan, & O'Brian, 1994).

Virus‐induced gene silencing (VIGS) technology is a reverse genetic technique that has been widely used to study biological function of plant genes (Romero, Tikunov, & Bovy, 2011). Since its introduction in 1995, VIGS has been applied in about 30 different species, including *A. thaliana*,* N. tabacum*, and *Solanum lycopersicon* (Becker & Lange, 2010). Despite its successful application in many species, VIGS efficiency depends on the distribution and the movement of the virus in plants (Romero et al., 2011). In consequence, it was recommended to use an additional reporter gene such as phytoene desaturase (PDS) to estimate the level of gene silencing (Senthil‐Kumar et al., 2007).

VIGS development requires many steps (Becker & Lange, 2010). In the first step, sequences corresponding to the host gene to be silenced are inserted into the viral genome. After creation of the engineered virus, it is inoculated in the host plant. Plant infection with the engineered virus initiates the synthesis of the viral dsRNA. Dicerlike enzymes cleave the dsRNA into short interfering RNAs. These RNAs are recognizable by RNA‐induced silencing complex (RISC) and melted into single‐stranded RNAs (ssRNAs) which are afterward used as template to target gene degradation.

Because most viruses have a limited host range, numerous virus vectors have been developed for VIGS in plants, including potato virus X (PVX), tobacco mosaic virus (TMV), tomato golden mosaic virus (TGMV), tomato yellow leaf curl virus (TYLCV), and China virus satellite DNA (Igarashi et al., 2009). Although VIGS has been widely used to investigate gene functions in many herbaceous plant species, VIGS has rarely been used in woody plants. Tobacco rattle virus (TRV) was one of the first viruses devolved for VIGS in woody plants such as *Populus euphratica* and *P*. × *canescens* (Shen et al., 2015). In addition, *Citrus leaf blotch virus* (CLBV) has also been used to target the phytoene desaturase (*PDS*) gene in citrus plants (Agüero et al., 2014). Because some of the reliable VIGS vectors can induce similar symptoms to those caused by the silenced gene (Igarashi et al., 2009), it is necessary to develop more VIGS vectors for additional plant species, especially for woody crops. Development of more VIGS vectors will enhance plant genomic studies (Burch‐Smith, Anderson, Martin, & Dinesh‐Kumar, 2004).

*Citrus tristeza virus* (CTV) is a closterovirus which is transmitted by the brown citrus aphid (Bar‐Joseph, Marcus, & Lee, 1989), and it infects most commercial citrus varieties (Albiach‐Marti et al., 2004). T36 and T30 are the most predominant CTV strains in Florida (Folimonova, Robertson, Garnsey, Gowda, & Dawson, 2009; Harper, Cowell, Halbert, Brlansky, & Dawson, 2015). The CTV isolate T36 induces a decline in sweet orange trees grafted onto sour orange rootstock, but it does not induce any appreciable disease syndromes in other common commercial citrus hosts such as grapefruit, and even sweet orange on other rootstocks (Dawson, Robertson, Albiach‐Martí, Bar‐Joseph, & Garnsey, 2015). Folimonov, Folimonova, Bar‐Joseph, and Dawson (2007) developed a CTV strain T36‐based vector for transient expression of foreign genes in citrus trees using a green fluorescent protein (GFP) as a reporter. Hajeri, Killiny, El‐Mohtar, Dawson, and Gowda (2014) developed CTV strain T36‐based vector and demonstrated its capability in silencing transgene *GFP* in *Nicotiana benthamiana* line 16c and citrus endogenous gene *PDS* in citrus. The use of this mild T36 CTV vector offers many advantages: (a) It produces no symptoms in infected plants; (b) has been able to express foreign genes in citrus for many years (Dawson & Folimonova, 2013); (c) can be used for field application to protect against diseases or to treat infected plants (Hajeri et al., 2014); and (d) more than one foreign gene can be inserted into the CTV genome at the same time (Dawson & Folimonova, 2013; El‐Mohtar & Dawson, 2014).

In a recent study, we showed that wild‐type CTV‐T36 (CTV‐wt) does not produce dramatic effects on the phloem sap composition and released volatiles of *Citrus macrophylla* (Killiny, Hijaz, Harper, & Dawson, 2017). In the current study, we investigated the effects of silencing of the *ALAD* gene, using the CTV‐based silencing vector, on citrus leaf pigments, phytohormones, and volatile and non‐volatile metabolites. We hypothesize that silencing of *ALAD* will not only affect chlorophyll biosynthesis, but can also affect the synthesis of other pigments and metabolites.

## 2 EXPERIMENTAL PROCEDURES

### 2.1 Plant material

Alemow (*C. macrophylla*) plants were grown under controlled greenhouse conditions (temperature of 22–24°C, 16‐:8‐hr day–light cycle and 60% humidity) at the Citrus Research and Education Centre, Lake Alfred, FL. Plants were about 1 year old (approximately two feet tall and the stem of pencil thickness).

### 2.2 *Citrus tristeza virus*‐based vectors

**Figure 1:**
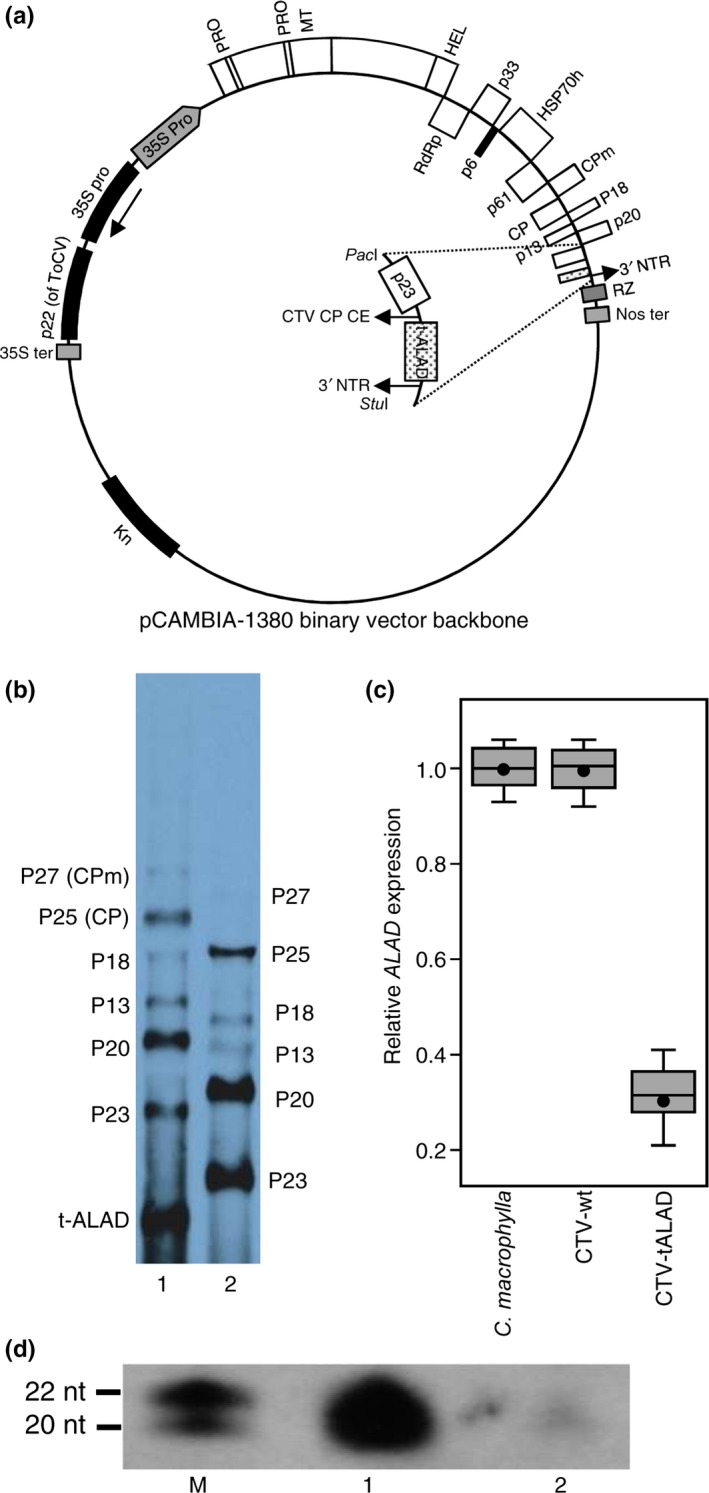
Silencing of *δ*‐aminolevulinic acid dehydratase (ALAD) using *Citrus tristeza* virus in a binary vector. (a) Schematic representation of full‐length infectious cDNA clones of *C. tristeza* virus (CTV) with its open reading frames (ORF) placed between enhanced 35S promoter of *Cauliflower mosaic* virus at the 5′end, ribozyme (RZ) of Subterranean clover mottle virus satellite RNA and nopaline synthase terminator (Nos ter) at the 3′end in the binary vector pCAMBIA‐1380. (b) Northern blot shows the subgenomic RNAs (sgRNAs) of CTV and an extra sgRNA for tALAD, accumulated in CTV‐tALAD plants (1) compared to CTV‐wt plants (2). The blot was hybridized with digoxigenin labeled minus‐sense ribo‐probe specific to the 3′nontranslated region of CTV. (c) Gene expression of *ALAD in CTV‐tALAD*,* CTV‐wt*, and *Citrus macrophylla*. Elongation factor 1‐alpha (*EF1*), F‐box/kelch‐repeat protein (*F‐box*), glyceraldehyde‐3‐phosphate dehydrogenase GAPC1, cytosolic (*GAPC1,* also known as *GAPDH*), and SAND family protein (*SAND*) were used as endogenous gene (reference gene) to normalize the data of gene expression. Horizontal thick lines indicate the medians, black dots indicate the means, boxes show the interquartile ranges including 25%–75% of the values, and whiskers reflect the highest and the lowest value of data. Experiments in triplicate are presented. (d) Accumulation of ALAD‐specific small interfering RNAs (siRNAs) in CTV‐tALAD plants (1) compared to CTV‐wt (2)

The infectious cDNA clones of CTV isolate T36; GenBank accession no. AY170468) in the binary vector pCAMBIA‐1380 (Figure 1a) was used as a base plasmid for engineering all of the constructs used in this study (Hajeri et al., 2014). To clone the truncated *ALAD* gene (tALAD) and generate the CTV‐tALAD vector, primers were designed based on *Citrus sinensis ALAD* gene cDNA sequence (Phytozome Citrus Gene orange1.1g013861m.g). The antisense of the truncated fragment (400 nucleotides) of the *ALAD* gene was amplified using total RNA from *C. macrophylla* as a template by SuperScript^®^ III One‐Step RT‐PCR System with Platinum^®^ Taq DNA Polymerase (Life Technologies Corp.) and primers ALAD‐PacI.

(5′‐GAGC*TTAATTAA*TAAAAAGTGGATATACAAAATTTGCCGGTGAAAG‐3′) and ALAD‐StuI (5′‐GACG*AGGCCT*GCTTCGTCGGTATTTCACGCGCCTTGTG‐3′). The PCR product was digested with PacI and StuI restriction enzymes and cloned into similarly digested CTV‐tGFP by replacing tGFP with tALAD fragment.

Procedures for agroinfiltration of CTV constructs into *N. benthamiana* were followed as described previously (Hajeri et al., 2014). After 4–6 weeks postinfiltration, systemic leaves from *N. benthamiana* that tested positive for CTV by ELISA were harvested and used in isolating CTV virions and for inoculating *C. macrophylla* by the bark‐flap method as described previously (Hajeri et al., 2014). Five plants (about 1 year old) were inoculated with CTV‐tALAD virion, five with CTV‐wt, and five plants were left as controls. Inoculated plants were kept in the greenhouse until development of clear genotype (about 6 months). Large RNA northern blot hybridization, small RNA isolation, small RNA northern blot hybridization, reverse transcription quantitative PCR (RT‐qPCR) for plant tissue were performed as described previously (Hajeri et al., 2014).

### 2.3 High‐performance liquid chromatography analysis of citrus leaf pigments

At least three leaves were collected from each plant (five plants were sampled from each treatment, and each sample was analyzed twice). Leaves collected from each plant were placed together in a mortar, mixed with liquid nitrogen, ground into fine powder using a pestle, and citrus leaf pigments were extracted as described previously (Killiny & Nehela, 2017a). In short, about 0.1 g ground material was mixed with 400 μl of acetone 80% and 240 μl of ethyl acetate and vortexed for 30 s. Samples were left in the dark on ice for 10 min and vortexed twice followed by addition of 280 μl of water, and the mixture was centrifuged at 8,500 *g* for 5 min at 4°C. The organic upper layer was removed and dried under a nitrogen stream. The extracted pigments were resuspended in 200 μl ethyl acetate and analyzed immediately by high‐performance liquid chromatography (HPLC) as described previously by Wei et al. (2014a,b). The HPLC system consisted of an Agilent 1200 system with photodiode array detector (Agilent Technologies, Santa Clara, CA, USA). Chromatographic separation was performed using a C30 YMC carotenoid column, 250 × 4.6 mm I.D., S‐5 μm (YMC America, Allentown, PA, USA). The mobile phase composition and the gradient profile were as described by Mouly, Gaydou, Lapierre, and Corsetti (1999). The column temperature was set at 25°C, mobile phase flow rate was 1 ml/min, and injection volume was 20 μl. Absorbance of the various citrus leaf pigments and peak responses were monitored at several spectral wavelengths (230, 278, 350, 430 and 486 nm). The HPLC output data were analyzed using ChemStation software, B.03.02 (Agilent Technologies, CA, USA). Pigments were identified by comparing experimental retention times and UV–visible spectra with that of published literature (Mouly et al., 1999), as well as with authentic standards. Chlorophyll *a*, chlorophyll *b*,* α*‐carotene, *β*‐carotene, lutein and zeaxanthin were obtained from Sigma‐Aldrich (USA). A set of concentrations (1, 5, 10, 25, 50, and 100 ppm) were prepared from each standard. A 20 μl aliquot from each standard was injected into the HPLC under the same condition described above to establish calibration curves and calculate the concentrations of pigments in citrus leaves. The concentrations of the rest of pigments were estimated using the lutein calibration curve**.**

### 2.4 Analysis of leaf pigment by thin‐layer chromatography

A 10 μl aliquot of the extracted pigments was spotted on the silica thin‐layer chromatography (TLC) plate, and the pigments were separated using a mixture of n‐hexane‐acetone (70:30 v/v) as described by Zeb and Murkovic (2010).

### 2.5 Gene expression analysis

Total RNA was extracted using TRIzol^®^ reagent (Ambion^®^, Life Technologies, NY, USA). NanoDrop 2000 spectrophotometer (Thermo Scientific, USA) was used to estimate the quantity and quality of isolated RNA. For synthesizing cDNA, SuperScript first‐strand synthesis system (Invitrogen) with random hexamer primers was used as described in the manufacturer's instructions. SYBR^®^ Green PCR master mix (Applied Biosystems) was used to perform quantitative PCR (qPCR) on an ABI 7500 Real‐Time PCR System (Applied Biosystems). For each treatment, two technical replicates per biological replicate and five biological replicates per treatment were analyzed in triplicate. Primers for 48 genes were used to measure the gene expression. The 2^−ΔΔCT^ method was used to determine the relative expression of genes (Livak & Schmittgen, 2001). Elongation factor 1‐alpha (*EF1*), F‐box/kelch‐repeat protein (*F‐box*), glyceraldehyde‐3‐phosphate dehydrogenase GAPC1, cytosolic (*GAPC1*, also known as *GAPDH*), and SAND family protein (*SAND*) were used as endogenous genes (reference genes) to normalize the data of gene expression (Mafra et al., 2012; Wei et al., 2014a,b).

### 2.6 Leaves incubation in levulinic acid

Three young leaves from each plant (five plants from each treatment) were harvested, and each leaf was incubated separately by immersing its petiole in 1.5 ml of 100 mM levulinic in 10% dimethylsulfoxide solution (Beale & Castelfranco, 1974) for 0 and 6 hr at room temperature (20°C).

### 2.7 Extraction of *δ*‐ALA from citrus leaves

The *δ*‐ALA was extracted using 0.1 N HCl as described by Hijaz and Killiny (2016). In short, leaves were ground in liquid nitrogen and 0.2 g of the ground tissues was transferred to 2‐ml centrifuge tube. One millilitre of 0.1 N HCl was added, and the sample was vortexed for 2 min. After 10 min of incubation, samples were further vortexed. The procedure was repeated three times, and the sample was centrifuged at 22,000 *g* for 6 min at room temperature.

### 2.8 Colorimetric determination of *δ*‐ALA

The concentration of *δ*‐ALA in the 0.1 N HCl extract was determined colorimetrically as described by Mauzerall and Granick (1956). In short, 0.5 ml of the supernatant was transferred to a new centrifuge tube and was mixed with 0.5 ml of phosphate buffer (equal volume of 0.5 M NaH_2_PO_4_ and 0.5 Na_2_HPO_4_; pH: 6.8) and 0.1 ml ethyl acetoacetate. The mixture was placed in a boiling water bath for 10 min. Then, the sample was cooled to room temperature and centrifuged at 14,000 rpm for 6 min at room temperature. After centrifugation, 0.7 ml of the supernatant was mixed with an equal volume of modified Ehrlich's reagent containing 2N perchloric acid. The density of the pink color produced from the reaction of *δ*‐ALA‐pyrrole with Ehrlich's reagent was measured at 553 nm using a Shimadzu UV‐Vis spectrophotometer (UV‐1700 PharmaSpec (Torrance, CA, USA). Each biological sample was analyzed twice (two technical replicates). A set of *δ*‐ALA standards (10.0, 5.0, 2.5, 1.2, 0.6, and 0.0 ppm in water) was reacted with ethyl acetoacetate and Ehrlich's reagent as mentioned above, and results were used to construct the standard curve. The spectra of the standard and the samples were also scanned between 400 and 700 nm.

### 2.9 *δ*‐ALA derivatization for GC‐MS

The *δ*‐ALA‐pyrrole derivative was reacted with N‐methyl‐(N‐trimethylsilyl) trifluoracetamide (MSTFA) as described by Hijaz and Killiny (2016). In brief, a 0.4 ml of the hydrochloric acid extract was reacted with ethyl acetoacetate as mentioned above and *δ*‐ALA‐pyrrole derivative was extracted with 3 × 600 μl of ethyl acetate (Tomokuni & Ogata, 1972). Then, the ethyl acetate was evaporated under a nitrogen stream. A 40 μl of N‐methyl‐(N‐trimethylsilyl) trifluoroacetamide (MSTFA) reagent was added to the dried sample and was incubated at 85°C for 35 min. A 400 μl of 20 ppm *δ*‐ALA standard was derivatized in the same way and 1 μl of the derivatized standard or sample was injected into the GC‐MS running in the full scan mode as described by Hijaz and Killiny (2016).

### 2.10 Colorimetric determination of starch and sucrose

Starch and sucrose were extracted according to Cimò et al. (2013) with slight modifications. In short, about 100 mg of the same ground leaf tissues was mixed with 500 μl distilled water and vortexed for 30 s. The water extract was boiled for 10 min, vortexed for 10 s, and centrifuged at 650 *g* for 5 min. A 300 μl aliquot of the supernatant was mixed with 900 μl of 100% ethanol, vortexed for 10 s, and centrifuged at 18,000 *g* for 10 min. The supernatant was discarded, and the pellet was suspended in 1 ml distilled water and mixed with 50 μl of iodine solution. Starch was determined by monitoring the absorbance at 595 nm as described by Cimò et al. (2013) using rice starch as a standard. The absorbance was measured using a microplate spectrophotometer (Model 680, Bio‐Rad Laboratories, CA, USA). Sucrose determination was accomplished at 620 nm as described by van Handel (1968) using PharmaSpec ultraviolet 1700 spectrophotometer (Shimadzu Corporation, Japan). In short, a 100 μl aliquot of the supernatant was mixed with 100 μl 30% KOH and the mixture was boiled for 10 min. When samples cooled to room temperature, 3 ml of anthrone reagent was added and the samples were incubated at 40°C for 10 min for color development.

### 2.11 Analysis of carboxylic compounds and phytohormones in leaves by GC‐MS

Phytohormones and carboxylic compounds were extracted, from the same ground samples, as described previously (Nehela, Hijaz, Elzaawely, El‐Zahaby, & Killiny, 2016), and then derivatized with methyl chloroformate after spiking of each sample with 5 μl aliquot of 200 ppm heptadecanoic acid, which is not found in citrus leaves, as internal standard. Phytohormones and carboxylic acids were analyzed as previously reported (Killiny & Nehela, 2017b; Nehela et al., 2016). Identification of metabolites was further confirmed by comparing their retention time, linear retention indices (LRIs), and mass spectra with authentic standards. Compound peak areas were normalized to the internal standards (heptadecanoic acid). Quantification of metabolites was based on the peak areas obtained from a series of reference standards derivatized and injected under the same conditions as samples. Calibration curves were constructed from the linear regressions obtained by plotting the concentration versus peak area for each standard.

### 2.12 Collection of volatile organic compounds from intact citrus leaves

Released leaf volatiles from intact, young intermediate, and mature leaves (hardened) from branches of *C. macrophylla* were collected on a mixed SPME fiber (50/30 μm divinylbenzene/carboxen/polydimethylsiloxane, 1‐cm fiber, Supelco) and analyzed using GC‐MS as described by Killiny and Jones (2017). Wiley 9th ed., NIST 2011, and Wiley Flavor and Fragrance mass spectral libraries were used for volatile identification as well as by comparing spectra to standards when available. Volatile standard was prepared in n‐hexane and was run using the same GC temperature program as samples. Five plants were sampled from each treatment, and three different types of leaves (young, intermediate, and mature) were taken from each plant.

### 2.13 Statistical analysis

For HPLC and GC‐MS, five biological and two technical replicates per treatment were analyzed. Comparison of pigment concentrations among the three different treatments was performed using the analysis of variance (ANOVA), followed by post hoc pairwise comparisons using a Tukey–Kramer honestly significant difference test (Tukey's HSD). Statistical significance was established as *p*‐value <0.05. The level of other metabolites in CTV‐tALAD plants was compared to that of CTV‐wt (T36) plants using two‐tailed *t* test, and statistical significance was established as *p*‐value <0.05. Two‐way hierarchical cluster analysis (HCA) was also performed with the means of the gene expressions for each treatment. Distance and linkage were measured using the Bray–Curtis similarity measure method (Michie, 1982). 3D‐surface plots were performed among the three pigment groups (chlorophylls, carotenes, and xanthophylls) using the total concentration of each group.

## 3 RESULTS

### 3.1 CTV‐tALAD induced gene silencing and produced yellow islands and necrosis in citrus

Northern blot analysis of RNA showed a of the extra subgenomic RNA in CTV‐tALAD plants compared to CTV‐wt plants (Figure 1b). In addition, RT‐qPCR showed a twofold to threefold downregulation of *δ*‐ALAD mRNA in CTV‐tALAD plants compared to CTV‐wt (Figure 1c). The northern blot also showed high accumulation of ALAD‐specific small interfering RNAs (siRNAs) in CTV‐tALAD plants compared to CTV‐wt (Figure 1d).

**Figure 2:**
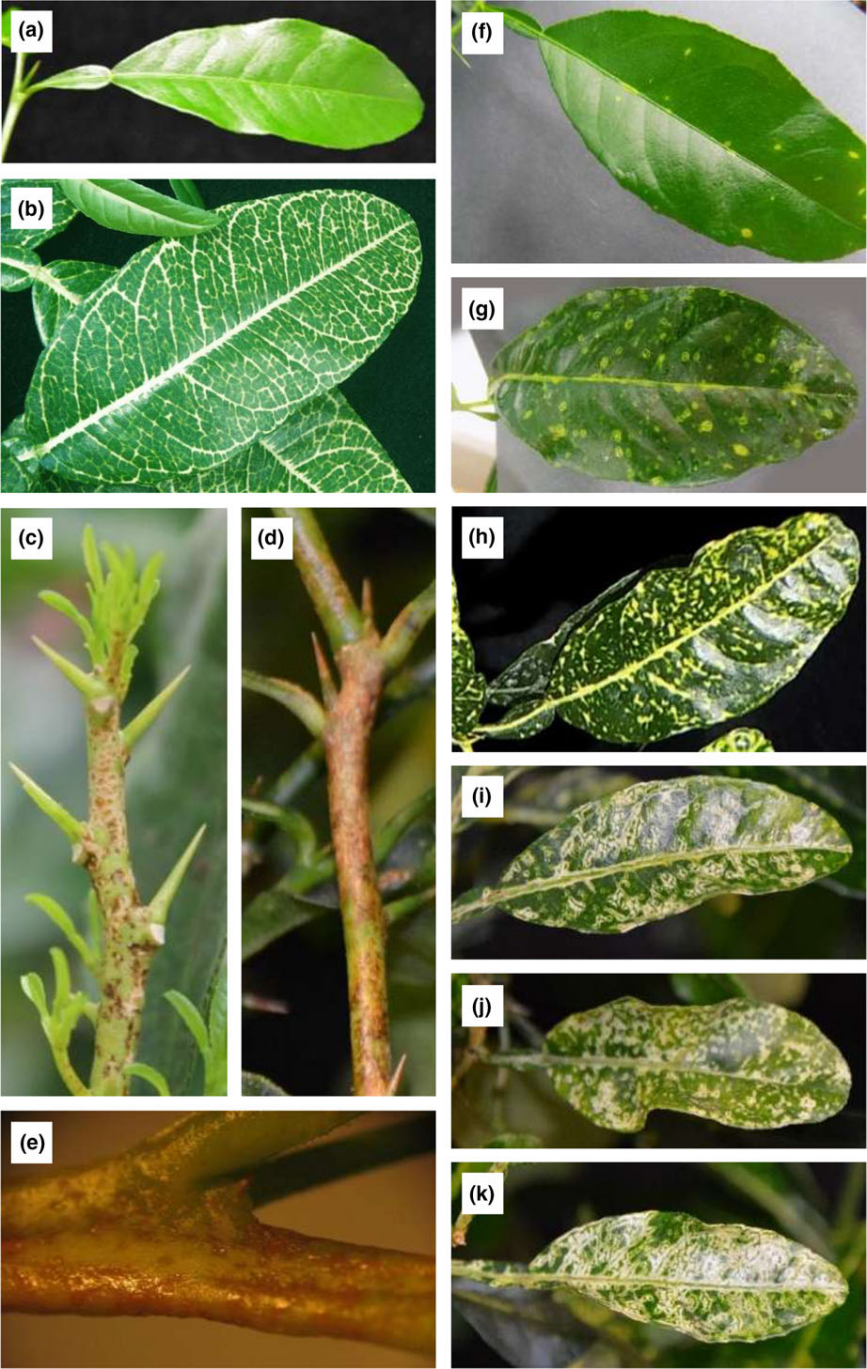
Phenotype observed in CTV‐tALAD plants and CTV‐tPDS plants. (a) CTV‐wt. (b) Photobleaching in CTV‐tPDS plants. (c–e) Yellow islands and necrosis in apical growing and stem of CTV‐tALAD plants. (f–k) Development of yellow islands and necrosis in leaves of CTV‐tALAD

*Citrus macrophylla* plants inoculated with CTV‐tALAD virions displayed yellow islands and necrosis in leaves, thorns and stems, and the apical meristem of citrus plants (Figure 2c–k). No yellow islands or necrosis was observed in CTV‐wt plants (Figure 2a). The symptoms start as a few dots in developing young leaves and the number of dots and their intensity grows until they cover most of the leaf surface (Figure 2f–k). The ALAD phenotype was also different from the PDS‐silenced plants (Figure 2b). The PDS plants showed a photobleaching phenotype (Figure 2b), whereas the CTV‐tALAD plants showed yellow islands and necrosis in leaves, stems, and the apical meristem (Figure 2c–f).

### 3.2 Silencing of *ALAD* reduced chlorophyll content and altered the levels of carotenoids

**Figure 3:**
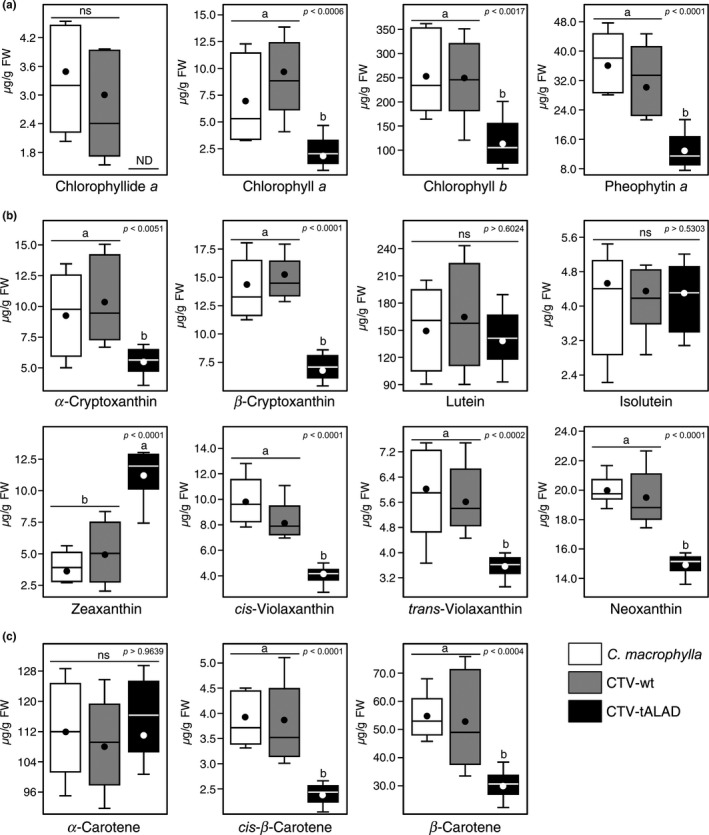
Concentrations (μg/g FW) of leaf pigments from control, CTV‐wt, and CTV‐tALAD *Citrus macrophylla* plants using HPLC (*n* = 10). Horizontal thick lines indicate the medians, black dots indicate the means, boxes show the interquartile ranges including 25%–75% of the values, whiskers reflect the highest and the lowest value of data. Different letters indicate statistically significant differences among treatments (*p* < 0.05), while “ns” indicates no significant differences among treatments using a Tukey–Kramer honestly significant difference test (Tukey's HSD)

Fifteen pigments were identified in the leaf extract using HPLC‐PDA (Supporting Information Figure S1a), whereas only eight of these pigments were identified using TLC (Supporting Information Figure S1b). The CTV‐tALAD significantly reduced the levels of chlorophyll *a*, chlorophyll *b*, chlorophyllide *a*, and pheophytin *a* compared to the controls (Figure 3). The reduction in chlorophyll *a*, chlorophyll *b*, and pheophytin in CTV‐tALAD plants was observed in the TLC results (Supporting Information Figure S1b).

While *α*‐ and *β*‐cryptoxanthin, *cis‐* and *trans*‐violaxanthin, and neoxanthin were reduced in CTV‐tALAD plants, zeaxanthin was increased (Figure 3). The *β*‐carotene and the *cis‐β*‐carotene were also significantly reduced in CTV‐tALAD plants (Figure 3). Lutein, isolutein, and *α*‐carotene were not significantly affected in CTV‐tALAD plants (Figure 3). The TLC also showed that violaxanthin and carotenes were reduced in CTV‐tALAD plants (Supporting Information Figure S1b).

**Figure 4:**
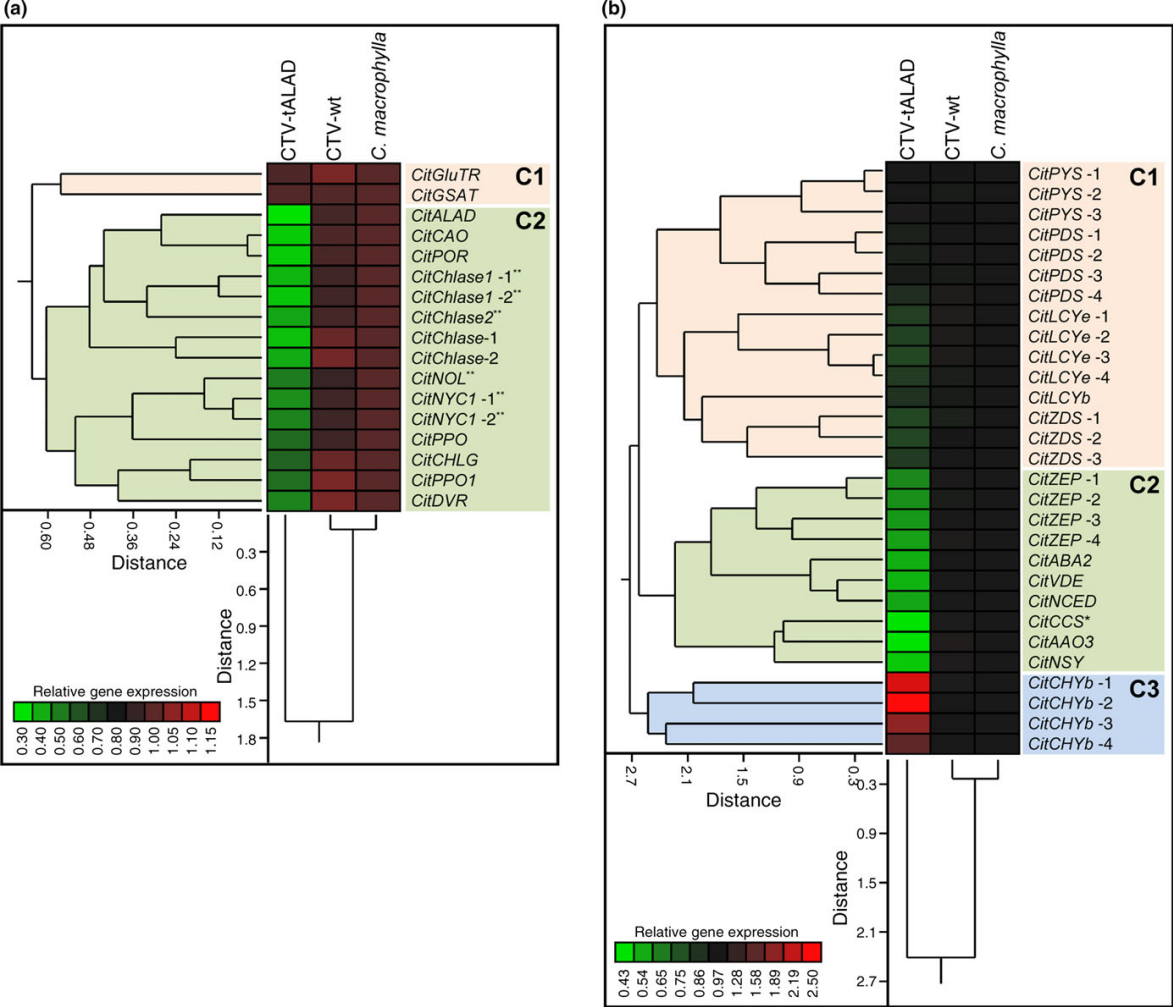
Differential biosynthetic gene expression patterns of chlorophylls (a) and carotenoids (b) and detected in leaves of CTV‐tALAD, CTV‐wt, and *Citrus macrophylla*. Rows represent the genes while columns represent the treatments (*n* = 30). Lower expressions levels are colored green, and higher expressions are colored red. Treatments and genes were organized using two‐way HCA based on similarities in autoscaled values and correlations, respectively. The full lists of expressed genes, accession numbers, and primers are available in our previous study (Killiny & Nehela, 2017a). See text for full names

To investigate the effect of silencing of the *ALAD* gene on the other genes implicated in the biosynthesis pathway of leaf pigments, the transcription levels of 46 genes involved in biosynthetic pathways of chlorophylls (17 genes) and carotenoids (29 genes) were investigated in *C. macrophylla*, CTV‐wt, and CTV‐tALAD plants (Figure 4). Gene expression data were normalized using four reference genes (*EF1*,* F‐box*,* GAPDH*, and *SAND*). These genes showed high stability and were used for transcript normalization in different citrus organs under biotic stress (Mafra et al., 2012; Wei et al., 2014a,b). The normalizing expression levels using the four reference genes were very similar to each other.

The presented heatmap and the hierarchical clustering dendrogram (HCD) between chlorophyll biosynthetic genes in Figure 4a show that all chlorophyll biosynthetic genes were separated into two major clusters. The first cluster (C1; about 0.55 distance) includes only two genes, glutamyl‐tRNA reductase 1 (*CitGluTR*) and glutamate‐1‐semialdehyde 2,1‐aminomutase (*CitGSAT*), which were not affected in CTV‐tALAD plants compared to the two controls (*C. macrophylla* and *C. macrophylla* with CTV‐wt). The second cluster (C2; about 0.60 distance) contains 15 genes, which were downregulated in CTV‐tALAD plants compared to the controls. These include *δ*‐aminolevulinic acid dehydratase 1 (*CitALAD*), chlorophyllide *a* oxygenase (*CitCAO*), protochlorophyllide reductase (*CitPOR*), chlorophyllase (*CitChlase*s, also known as *CLH*s), chlorophyll(ide) *b* reductase (*CitCBR*s), including chlorophyll(ide) *b* reductase ‐NON‐YELLOW COLORING 1 (*CitNYC1*) and chlorophyll(ide) *b* reductase ‐NYC1‐Like (*CitNOL*), protoporphyrinogen oxidase (*CitPPO*), chlorophyll synthase (*CitChlG*), and divinyl chlorophyllide a 8‐vinyl‐reductase (*CitDVR*) (Figure 4a). Furthermore, HCD among treatments indicated that while CTV‐wt plants were very similar to *C. macrophylla* plants (less than 0.1 distance), CTV‐tALAD plants clustered separately (about 1.65 distance) (Figure 4a).

In addition, silencing of the *ALAD* gene altered the expression of several genes implicated in carotenoids biosynthesis pathways. Of the twenty‐nine selected genes, ten genes were downregulated, whereas only four genes were upregulated compared to the controls (Figure 4b). Likewise, the HCA revealed that all carotenoid biosynthetic genes were clustered in three main clusters. The first cluster (C1; about 2.45 distance) included 15 unaffected genes, including phytoene synthase (*CitPYS*s), phytoene desaturase (*CitPDS*s), lycopene *ε*‐cyclase (*CitLCYe*s), lycopene *β*‐cyclase (*CitLCYb*), and *ζ*‐carotene desaturase (*CitZDS*s) (Figure 4b). The second cluster (C2; about 2.25 distance) included ten downregulated genes implicated in the ABA‐biosynthetic pathway, including zeaxanthin epoxidase (*CitZEP*s), short‐chain alcohol dehydrogenase (*CitABA2*), violaxanthin de‐epoxidase (*CitVDE*), putative 9‐*cis*‐epoxycarotenoid dioxygenase 3 (*CitNCED*), capsanthin/capsorubin synthase (*CitCCS*), abscisic aldehyde oxidase (*CitAAO3*), and neoxanthin synthase (*CitNSY*) (Figure 4b). The third cluster included four isoforms of carotenoid hydroxylase *β*‐ring (*CitCHYb*s; converts *β*‐carotene to *β*‐cryptoxanthin and then zeaxanthin), which were upregulated in CTV‐tALAD plants compared to the controls (Figure 4b). Furthermore, the HCA among treatments in Figure 4b shows that CTV‐wt plants were closer to *C. macrophylla* (less than 0.3 distance) than to CTV‐tALAD (about 2.4 distance).

**Figure 5:**
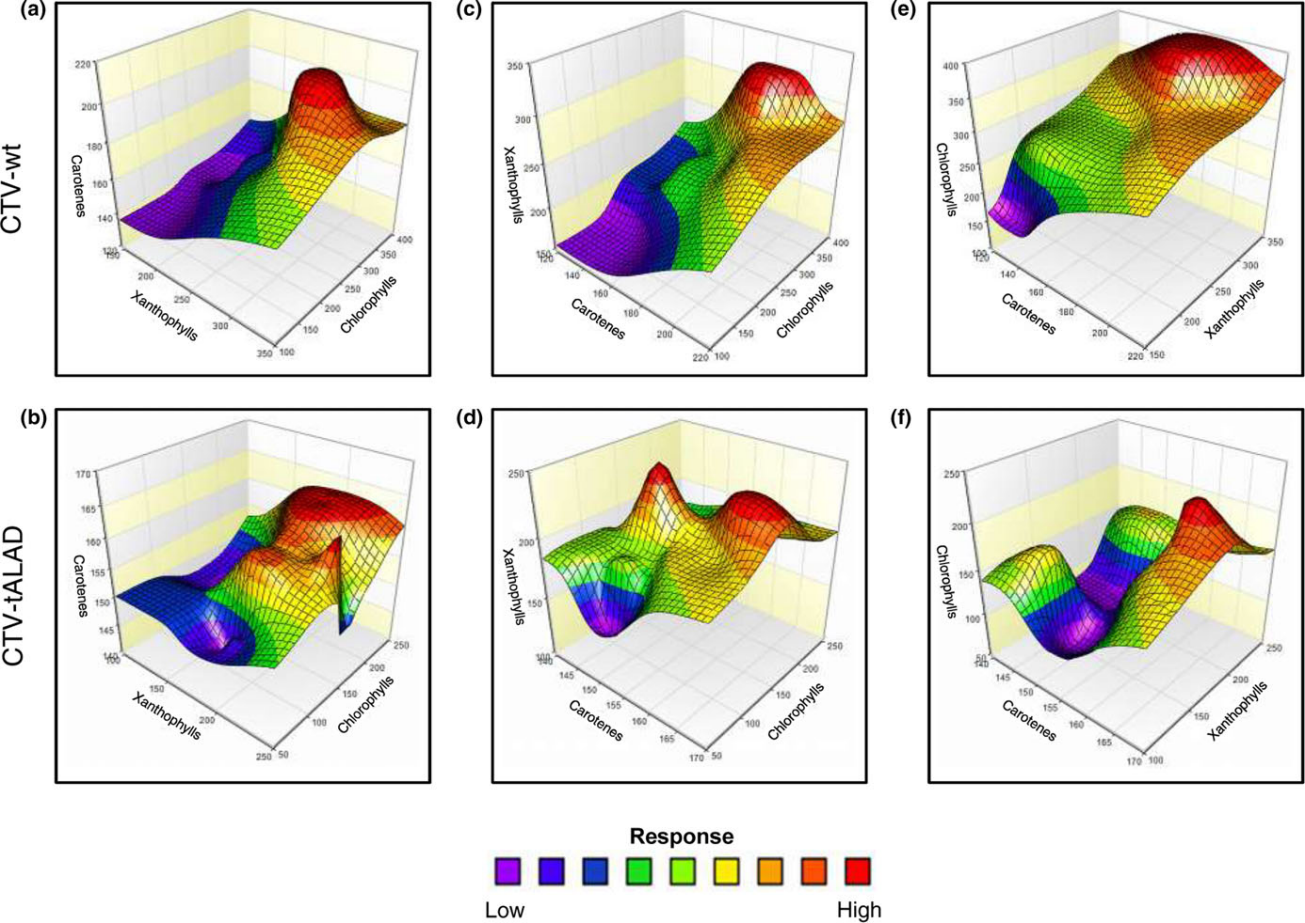
Three‐dimensional surface plots of leaf pigments groups detected using HPLC in leaves from CTV‐wt and CTV‐tALAD plants. (a,b) The reciprocal interactions of chlorophylls and xanthophylls on carotene content. (c,d) The reciprocal interactions of carotenes and chlorophylls on xanthophyll content in different treatments. (e,f) The reciprocal interaction of carotenes and xanthophylls on chlorophyll content

As no differences were found in the concentration of studied pigments and their biosynthetic genes between the *C. macrophylla* and *C. macrophylla* infected with CTV‐wt, we chose to use CTV‐wt as the control in all subsequent analyses. The 3D surface plots were obtained using the whole data matrix to understand the relationships between chlorophyll, xanthophyll, and carotene groups in CTV‐wt and CTV‐tALAD plant leaves (Figure 5a–f). Overall, the silencing of the *ALAD* gene affected the total net profiles (TNPs) of other pigments, causing a complex relationship among different pigments groups. The effect of xanthophyll and chlorophyll (as two input parameters) on carotene content (as an associated performance metric) is presented in Figure 5a,b. The carotenes TNP of CTV‐tALAD was totally different compared to CTV‐wt plants. While the carotenes TNP of CTV‐wt plants was flatter and had a clear peak in high chlorophylls and high xanthophylls (Figure 5a), the carotenes TNP of CTV‐tALAD plants had more plateaus and curvatures without any clear peaks (Figure 5a). Furthermore, the effect of carotene and chlorophyll content on xanthophyll content is presented in Figure 5c,d. The xanthophylls TNP of CTV‐tALAD plants was very different compared to CTV‐wt, which was flatter with only one small peak under the high‐carotene and high‐chlorophyll conditions (Figure 5c). The xanthophylls TNP of CTV‐tALAD plants had a clear curvature at relatively low levels of carotenes and moderate levels of chlorophylls with a very sharp peak at relatively low‐carotene and moderate‐chlorophyll conditions (Figure 5d). In addition, the effect of xanthophylls and carotenes on chlorophyll content presented in Figure 5e,f. As observed, the TNP of chlorophyll content in CTV‐tALAD plants appeared very different from that of CTV‐wt plants, which was flatter with a small plateau (Figure 5e), while the chlorophylls TNP of CTV‐tALAD plants had two clear curvatures: the first one at relatively low levels of carotenes and moderate levels of xanthophylls, and the second one at low levels of carotenes and high levels of xanthophylls. In addition, it had a clear peak at high‐carotene and high‐xanthophyll conditions (Figure 5f).

### 3.3 CTV‐tALAD plants accumulated *δ*‐ALA in developing leaves

**Figure 6:**
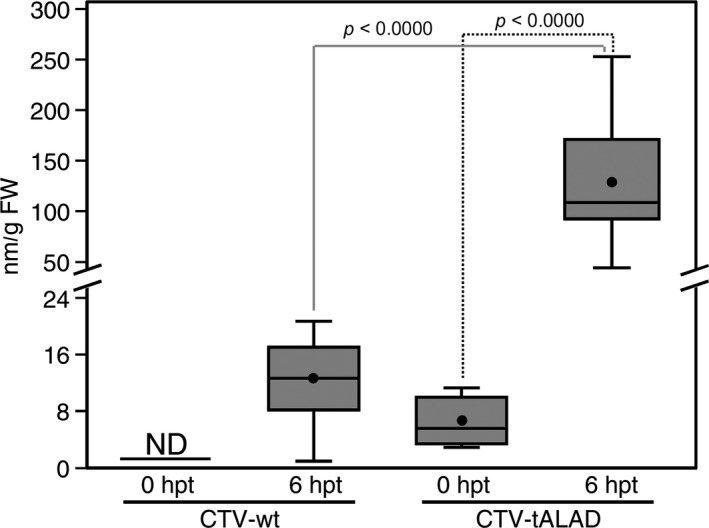
Colorimetric determination of *δ*‐ALA. Level of *δ*‐ALA in developing young leaves from CTV‐wt and CTV‐tALAD plants (*n* = 5) after 0‐ and 6‐hr incubation with 100 mM levulinic acid. *p*‐value <0.05 indicates statistically significant differences among treatments using two‐tailed *t* test

The colorimetric assay showed that *δ*‐ALA accumulated in the developing leaves from CTV‐tALAD (6.6 ± 0.5 mg/g FW) plants, whereas no *δ*‐ALA was detected in the CTV‐wt (Figure 6). No *δ*‐ALA accumulation was detected in mature leaves from CTV‐tALAD or CTV‐wt plants. In addition, the amount of *δ*‐ALA detected in CTV‐tALAD plants after incubation with levulinic acid was higher (138 ± 51 mg/g FW) than that (11 ± 5 mg/g FW) detected in the CTV‐wt after 6‐hr incubation with levulinic acid (Figure 6). The spectra of the pink color developed in the leaf extracts from CTV‐tALAD plants (without incubation with levulinic acid) were similar to those obtained using *δ*‐ALA standard and leaf extracts from leaves incubated with levulinic acid (Supporting Information Figure S2a). The GC‐MS results also showed that CTV‐tALAD plants accumulate more *δ*‐ALA when incubated with levulinic acid than the CTV‐wt (Supporting Information Figure S2b). The mass spectra of the *δ*‐ALA detected in CTV‐tALAD plants and in the controls after incubation in levulinic acid were similar to those obtained with authentic standard (Supporting Information Figure S2c).

### 3.4 Silencing of *ALAD* decreased the level of photosynthates and affected citrus leaf metabolites and phytohormones

**Figure 7:**
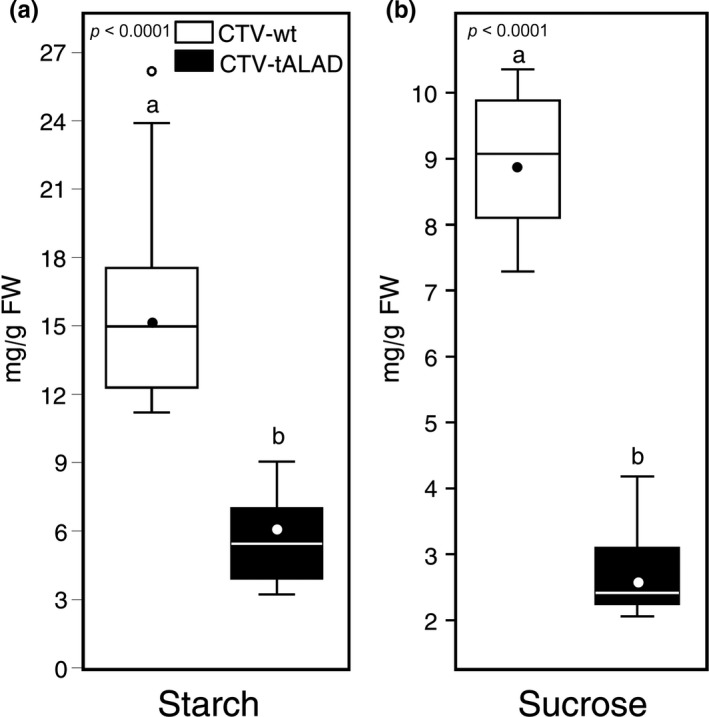
Concentrations (mg/g FW) of starch (a) and sucrose (b) in leaves of CTV‐wt and CTV‐tALA, plants using HPLC (*n* = 10). Horizontal thick lines indicate the medians, black dots indicate the means, boxes show the interquartile ranges including 25%–75% of the values, and whiskers reflect the highest and the lowest value of data. *p*‐value <0.05 indicates statistically significant differences among treatments using two‐tailed *t* test

The starch level was significantly reduced (about fivefold) in CTV‐tALAD plants compared to the controls (Figure 7a). The sucrose level was also reduced (about threefold) by CTV‐tALAD infection (Figure 7b). No effects were observed on the level of sucrose or starch in CTV‐wt.

A few amino acids including l‐alanine, *gamma*‐aminobutyric acid (GABA), l‐threonine, l‐glutamic, and l‐phenylalanine significantly decreased in leaves of CTV‐tALAD plants, whereas l‐asparagine increased (Table 1). None of the detected fatty acids was affected in the leaves of CTV‐tALAD plants, except linoleic acid, which was significantly increased (Table 1). Fumaric and succinic acid significantly increased in leaves of CTV‐tALAD plants (Table 1). In a similar manner, GABA, l‐threonine, and l‐phenylalanine decreased significantly in the apical meristems of CTV‐tALAD plants, whereas l‐asparagine increased significantly (Table 1). Linoleic and linolenic acid significantly increased in meristems of CTV‐tALAD plants, whereas oleic acid decreased significantly. Fumaric, succinic, and malic also increased significantly in the apical meristems of CTV‐tALAD plants (Table 1).

**Table 1:** Concentrations (ng/g FW) of different amino acids, organic acids, and fatty acids compounds detected in CTV‐wt and CTV‐tALAD leaves and apical meristems using GC‐MS (*n* = 5)

	Mature leaves		*t* test *p*‐value	Apical meristems		*t* test *p*‐value
	CTV‐wt	CTV‐tALA		CTV‐wt	CTV‐tALA	
Amino acids						
Glycine	22.4 ± 4.9	21.4 ± 1.7	0.6819	43.2 ± 2.2	43.3 ± 2.3	0.9442
l‐Alanine	612.5 ± 83.0	336.8 ± 85.2	0.0008	174.1 ± 70.6	165.6 ± 77.3	0.8605
l‐Valine	91.6 ± 7.4	97.7 ± 1.6	0.1417	85.8 ± 1.4	89.7 ± 3.1	0.0441
l‐Leucine	41.1 ± 0.9	42.6 ± 0.9	0.0303	37.3 ± 0.4	39.3 ± 0.8	0.0028
GABA	1288.7 ± 171.9	655.0 ± 172.2	0.0004	973.6 ± 57.6	799.0 ± 141.7	0.0486
l‐Isoleucine	54.7 ± 7.4	50.1 ± 2.4	0.2524	43.2 ± 0.6	42.7 ± 1.5	0.5286
l‐Threonine	142.6 ± 24.5	103.5 ± 12.2	0.0193	84.7 ± 10.2	67.8 ± 11.1	0.0372
l‐Proline	6787.8 ± 1362.0	6237.5 ± 1322.9	0.5351	4531.5 ± 972.5	4974.8 ± 473.5	0.3960
l‐Asparagine	3657.2 ± 1717.9	4164.1 ± 580.6	0.5599	947.6 ± 173.7	2260.2 ± 941.0	0.0343
l‐Aspartic acid	659.8 ± 60.8	526.8 ± 31.7	0.0049	242.1 ± 21.1	317.5 ± 35.5	0.0054
l‐Pyroglutamic acid	410.3 ± 85.2	400.7 ± 18.8	0.8166	14.3 ± 3.2	14.3 ± 10.8	0.9913
l‐Serine	1588.4 ± 239.5	1692.8 ± 201.7	0.4780	869.8 ± 151.7	717.3 ± 252.5	0.2874
l‐Glutamic acid	2115.4 ± 345.7	1417.7 ± 160.3	0.0073	469.3 ± 263.0	462.8 ± 194.5	0.9659
l‐Phenylalanine	164.1 ± 20.2	113.6 ± 14.2	0.0024	151.9 ± 14.8	75.3 ± 27.5	0.0014
l‐Lysine	216.1 ± 9.9	214.1 ± 9.2	0.7580	208.5 ± 2.0	209.2 ± 2.8	0.6629
l‐Tyrosine	157.2 ± 72.4	122.9 ± 0.5	0.3495	120.8 ± 0.7	121.9 ± 3.0	0.4661
l‐Tryptophan	49.5 ± 1.1	48.2 ± 0.2	0.0452	57.2 ± 2.2	48.9 ± 2.4	0.0004
Total amino acids	18059.2 ± 3142.7	16245.3 ± 1958.6	0.3112	9054.9 ± 840.9	10449.8 ± 2033.5	0.2121
Fatty acids						
Palmitic acid (C16:0)	1172.6 ± 62.3	1235.6 ± 112.0	0.3121	1009.6 ± 99.0	1254.3 ± 256.6	0.1015
*α*‐Linolenic acid (C18:3)	10653.0 ± 2161.0	8767.2 ± 831.7	0.1264	5342.0 ± 331.6	6535.1 ± 588.8	0.0068
Linoleic acid (C18:2)	1780.6 ± 238.0	2276.9 ± 200.8	0.0077	684.6 ± 68.4	1647.2 ± 208.8	0.0002
Oleic acid (C18:1)	125.4 ± 55.2	88.3 ± 38.1	0.2550	121.6 ± 18.3	52.6 ± 28.7	0.0029
Stearic acid (C18:0)	279.9 ± 68.6	342.3 ± 50.3	0.1430	227.8 ± 17.9	368.0 ± 187.1	0.1694
Total fatty acids	14011.6 ± 2459.0	12710.3 ± 1079.1	0.3239	7385.6 ± 390.5	9857.1 ± 888.0	0.0017
Organic acids						
Fumaric acid	918.8 ± 30.6	981.2 ± 25.8	0.0086	817.7 ± 17.2	913.8 ± 39.7	0.0033
Succinic acid	506.1 ± 77.0	659.4 ± 61.3	0.0089	405.5 ± 10.0	628.0 ± 75.8	0.0025
Malic acid	2817.3 ± 319.0	3117.9 ± 283.7	0.1544	1574.9 ± 168.7	2202.5 ± 228.9	0.0015
Quinic acid	1548.1 ± 101.2	1497.5 ± 292.0	0.7296	1639.6 ± 322.6	1488.9 ± 745.3	0.6939
Citric acid	1846.7 ± 42.7	1851.8 ± 189.8	0.9554	1759.1 ± 52.3	1724.9 ± 60.3	0.3668
Ferulic acid	390.5 ± 189.7	503.5 ± 84.8	0.2732	747.6 ± 225.7	149.6 ± 30.0	0.0038
Total organic acids	8027.4 ± 376.9	8611.4 ± 678.2	0.1413	6944.4 ± 744.2	7107.7 ± 960.1	0.7718

*Note*

Means were considered significantly different if *p*‐value was less than 0.05.

**Figure 8:**
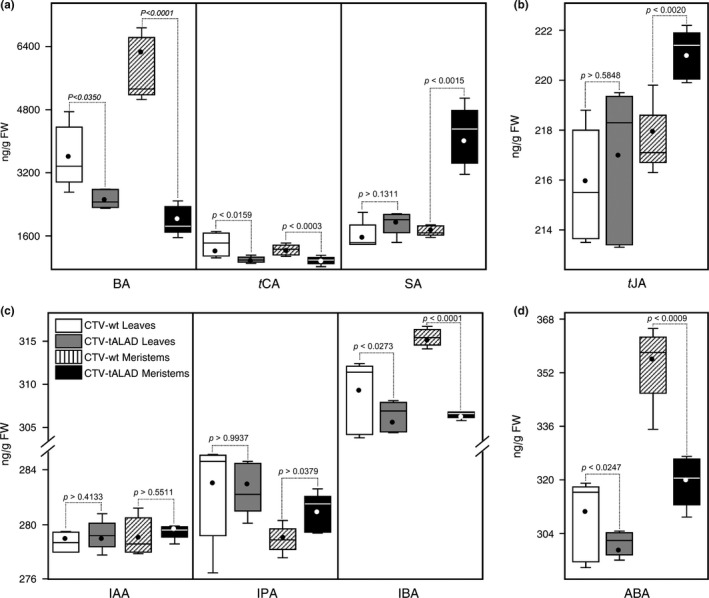
Concentrations of different phytohormones and BA, *t*CA in leaves and apical meristems of CTV‐wt and CTV‐tALA plants a using GC‐MS‐SIM (*n* = 10). (a) BA, *t*CA, and SA. (b) *t*JA, (c) auxins. (d) ABA. Horizontal thick lines indicate the medians, black/white dots indicate the means, boxes show the interquartile ranges including 25%–75% of the values, and whiskers reflect the highest and the lowest number of data. *p*‐value <0.05 indicates statistically significant differences among treatments using two‐tailed *t* test. ABA: abscisic acid; BA: benzoic acid; IAA: indole‐3‐acetic acid; IBA: indole‐3‐butyric acid; IPA: indole‐3‐propionic acid; SA: salicylic acid; *t*CA: *trans*‐cinnamic acid; *t*JA: *trans*‐jasmonic acid

Levels of phytohormones and their precursors were affected in leaves of CT‐tALAD plants. BA, *t*CA, IBA, and ABA decreased in leaves from CTV‐tALAD plants, whereas SA, JA, *t*JA, IAA, and IPA were not affected (Figure 8a–d). The levels of BA, *t*CA, IBA, and ABA were also reduced in the apical meristems of CTV‐tALAD plants, whereas SA, *t*JA, and IPA increased significantly (Figure 8a–d).

The emission of volatile organic compounds was mostly unaffected in juvenile leaves of CTV‐tALAD plants. Likewise, intermediate‐aged leaves were relatively unaffected, except for (*E*)‐*β*‐ocimene, (*E*)‐*α*‐bergamotene, (*E*)‐*β*‐farnesene, and valencene which increased dramatically (Table 2). Most of the observed differences between the mature leaves of the CTV‐wt and the CTV‐tALAD plants were small but significant, including increases in *β*‐myrcene, (*E*)‐*β*‐ocimene, allo‐ocimene, and (*Z*)‐*β*‐caryophyllene (Table 2).

**Table 2:** The relative amounts of volatiles collected from leaves of CTV‐tALA plants compared to the CTV‐wt (T‐36), (*n = 5*)

Compound	RT	Young leaves		Intermediate leaves		Mature leaves	
		ALAD/T36	*t* test	ALAD/T36	*t* test	ALAD/T36	*t* test
*α*‐Pinene^a^	3.9	0.6	0.4451	1.3	0.5812	0.5	0.0974
*β*‐Myrcene^a^	4.5	0.6	0.4034	1.4	0.4190	0.5	0.0268
*β*‐Phellandrene^a^	5.1	0.4	0.2111	0.3	0.2879	0.3	0.1136
Limonene^a^	5.2	0.6	0.1294	0.7	0.3473	0.8	0.2355
(*E*)‐*β*‐Ocimene^a^	5.3	14.4	0.0535	24.2	0.0002	11.0	0.0213
*γ*‐Terpinene^a^	5.4	0.7	0.4730	1.0	0.9415	0.5	0.1025
*α*‐Terpinolene^a^	5.8	0.5	0.3827	1.2	0.5650	0.3	0.0608
Linalool^a^	6.0	1.5	0.5709	0.7	0.6556	0.6	0.2731
Perillene	6.2	0.5	0.3189	2.3	0.3729	1.2	0.8031
*allo*‐Ocimene	6.4	0.1	0.0831	0.1	0.0439	0.0	0.0105
Limonene oxide	6.6	6.5	0.2807	0.4	0.5352	5.3	0.3174
Citronellal^a^	6.7	0.9	0.8148	0.3	0.2484	0.6	0.4206
Methyl salicylate^a^	7.4	1.1	0.8225	0.4	0.4109	0.5	0.3032
Decanal^a^	7.5	0.7	0.3810	0.3	0.1617	0.7	0.4465
Nerol^a^	7.6	0.4	0.2950	0.6	0.5175	0.3	0.1433
Neral	7.7	4.4	0.2855	2.2	0.2510	4.3	0.1757
Geraniol^a^	7.9	1.3	0.6924	0.5	0.5468	0.7	0.4871
Geranial^a^	8.3	0.8	0.6808	0.4	0.4089	0.7	0.4392
*δ*‐Elemene^a^	9.3	5.5	0.0561	8.8	0.0508	7.1	0.1312
Citronellyl acetate	9.4	0.0	0.2659	0.3	0.3007	2.4	0.4045
*α*‐Cubebene	9.5	10.3	0.1283	3.6	0.0543	10.9	0.1176
Geranyl acetate^a^	9.8	1.4	0.6686	0.6	0.6370	1.8	0.3424
*β*‐Cubebene	9.9	0.3	0.1275	0.2	0.2761	0.2	0.0725
*β*‐Selinene	10.0	1.2	0.8040	0.8	0.8353	0.9	0.7858
(*Z*)‐*β*‐Caryophyllene	10.4	0.1	0.0617	0.1	0.2308	0.1	0.0177
(*E*)‐*β*‐Caryophyllene	10.5	1.0	0.9743	1.0	0.9567	0.7	0.4982
(*E*)‐*α*‐Bergamotene	10.6	207.4	0.1002	104.9	0.0000	82.7	0.0616
(*E*)‐*β*‐Farnesene^a^	10.7	5.5	0.2186	8.7	0.0006	9.9	0.1174
*α*‐Humulene^a^	11.0	1.2	0.8717	0.6	0.5649	3.7	0.4121
*α*‐Selinene	11.3	1.1	0.8653	0.5	0.4726	0.4	0.1386
Valencene^a^	11.5	3.3	0.0386	11.2	0.0018	4.8	0.1414
*β*‐Bisabolene	11.5	1.1	0.8789	1.2	0.7336	1.2	0.7178
Cadinene	11.7	3.4	0.3375	2.2	0.1478	2.1	0.3136
Elemol	12.0	2.4	0.1686	3.6	0.1237	3.3	0.2017
*γ*‐Gurjunene	12.2	0.2	0.2141	4.5	0.1795	0.4	0.1831
Caryophyllene oxide	12.5	2.1	0.4266	2.6	0.0145	0.9	0.7639
Methyl jasmonate^a^	13.1	2.9	0.2930	1.2	0.7969	0.8	0.6841

*Notes*

The relative amounts were calculated by dividing the peak area of each compound in the CTV‐tALAD plants on the peak area of the same compound in the CTV‐wt (T‐36).

a Compounds were confirmed by comparing their retention time and mass spectra with authentic standards. Means were considered significantly different if *p*‐value was less than 0.05.

## 4 DISCUSSION

*δ*‐aminolevulinic acid dehydratase is a key enzyme in plants and is required for the synthesis of all classes of tetrapyrroles, which play important roles in many biological processes, including photosynthesis and respiration. In consequence, we hypothesized that inhibition of this enzyme would not only have an impact on chlorophyll biosynthesis and photosynthesis, but also affect other pigments and could lead to changes in both primary and secondary metabolites. Therefore, we carried out targeted and nontargeted metabolic analysis on CTV‐tALAD plants. The level of *δ*‐ALA was determined to confirm the successful silencing of the ALAD gene. We evaluated chlorophyll content as accumulation of *δ*‐ALA was expected to reduce chlorophyll biosynthesis. In addition, we analyzed carotenoid content because carotenoids are functionally linked to chlorophyll in photosynthetic light harvesting. Reduction in chlorophyll biosynthesis was expected to decrease the rate of photosynthesis; thus, we determined the level of the main photosynthates (sucrose and starch). CTV‐tALAD plants showed clear symptoms of necrosis, which indicated they were under stress; therefore, four different groups of phytohormones including the main growth regulators and stress‐associated groups (auxins, salicylates, jasmonates, and ABA) were quantified. At last, we performed nontargeted metabolomic analysis on volatile and nonvolatile compounds to get more insights on the metabolic status of CTV‐tALAD plants. Our results are discussed in the following paragraphs.

### 4.1 Silencing of *ALAD* accumulated *δ*‐ALA and reduced chlorophyll and photosynthates levels in citrus leaves

Silencing of *ALAD* led to the accumulation of *δ*‐ALA and reduction in chlorophyll and photosynthates in citrus leaves by the CTV‐based silencing vector. These results showed that ALAD is a critical enzyme in chlorophyll synthesis as it is required for the synthesis of all classes of tetrapyrroles (Tanaka & Tanaka, 2007). The *δ*‐ALA that is formed in developing tissues does not accumulate and is directly converted to PBG by ALAD (Kumar & Söll, 2000). Beale and Castelfranco (1974) showed that the rate of *δ*‐ALA accumulation in cucumber cotyledons treated with levulinic acid paralleled the rate of chlorophyll formation, and accumulation of *δ*‐ALA ceased when cucumber cotyledons were returned to dark (Beale & Castelfranco, 1974). In agreement with the previous results, our results also showed that the level of *δ*‐ALA in young leaves from CTV‐tALAD was higher than that in mature leaves, which is in agreement with the high rate of chlorophyll synthesis in young leaves.

Inhibition of *δ*‐ALA formation in mutant plants such as tobacco and Arabidopsis reduced chlorophyll and heme biosynthesis, but did not result in necrotic lesions (Tanaka & Tanaka, 2007). In agreement with these observations, our current study showed that silencing of *ALAD* also reduced chlorophyll *a,* chlorophyll *b*, chlorophyllide *a*, and pheophytin *a* synthesis and resulted in yellow islands. However, these changes were accompanied by necrosis in citrus plants. The gene expression results confirmed the reduced levels of pigments and showed that all selected genes in the chlorophyll biosynthesis pathway downstream of the silenced *ALAD* gene were downregulated. Our results indicated that accumulation of *δ*‐ALA could be more toxic to the plants than inhibition of its formation.

The reduction in chlorophyll indicated that CTV‐tALAD plants have a lower photosynthesis rate than control plants. In fact, our results showed that sucrose and starch levels (photosynthates) were reduced in CTV‐tALAD plants. The reduction in chlorophylls reduces the amount of absorbed light and harvested energy (ATP and NADPH); consequently, carbon fixation is reduced and results in a decrease in photosynthates in leaves.

### 4.2 Silencing of *ALAD* increased zeaxanthin level in citrus leaves

Interactions between chlorophylls and carotenoids play a major role in photosynthetic light harvesting. Besides absorbing light and harvesting energy, carotenoids, such as xanthophylls and carotenes, protect plants from light damage. In fact, absence of carotenoids in plants causes severe photo‐oxidation and leads to plant death (Bartley & Scolnik, 1995). Carotenoids reduce photo‐oxidation by quenching of chlorophyll triplets to prevent the formation of singlet oxygen that can oxidize lipids, proteins, and other components in plant cells (Bartley & Scolnik, 1995). The xanthophyll cycle plays an important role in photoprotection in plants (Demmig‐Adams, 1990). When the sunlight level exceeds the maximum that can be used by chlorophylls, plants can increase their zeaxanthin levels for photoprotection which in turn reduces chlorophyll fluorescence (Demmig‐Adams, 1990).

As the levels of chlorophylls were reduced in leaves from CTV‐tALAD plants, the amount of light that can be absorbed is expected to decrease. To prevent light damage, the CTV‐tALAD plants increased the level of zeaxanthin. These results were in agreement with the increase in the gene expression of the *CitLCHYB* genes, which are implicated in zeaxanthin synthesis. An increase in zeaxanthin and antheraxanthin and a decrease in violaxanthin were reported in *A. thaliana* after treatment with norflurazon, another specific competitive inhibitor of ALAD (Jung, 2004). The increase in the zeaxanthin was accompanied by an increase in nonphotochemical chlorophyll fluorescence quenching in norflurazon‐treated plants (Jung, 2004). In recent times, we also found that the reduction in chlorophyll levels in leaves from *C*Las‐infected sweet orange plants was also accompanied by an increase in zeaxanthin (Killiny & Nehela, 2017a). The increase in zeaxanthin indicated that reduction in chlorophyll as a result of silencing of *ALAD* induced light stress in CTV‐tALAD plants.

### 4.3 The phytohormonal profile indicated that CTV‐tALAD plants were under stress

Accumulation of *δ*‐ALA resulted in necrosis in CTV‐tALAD, indicating that these plants were under stress. In consequence, we investigated the level of the main phytohormones, which are associated with plant response to biotic and abiotic stresses. Our results showed that several phytohormones were affected by silencing of the *ALAD* gene. SA increased in the apical meristems of CTV‐tALAD plants. The *t*JA also increased in the apical meristems of CTV‐tALAD plants, and this increase was accompanied by an increase in its precursor, linolenic acid. An increase in *t*JA was observed in citrus plant after *Diaphorina citri* attack and this increase was also accompanied by an increase in linolenic acid and an upregulation of allene oxide synthase (AOS, a JA‐biosynthetic enzyme) (Nehela et al., 2018). SA and *t*JA are stress‐associated phytohormones in plants (Bari & Jones, 2009; (Nehela et al., 2018)). The SA pathway is associated with plant responses to biotrophic and hemibiotrophic pathogens and plays an important role in systemic acquired resistance (SAR) (Bari & Jones, 2009). On the contrary, the *t*JA pathway is associated with plant responses to necrotrophic pathogens and insect herbivory (Bari & Jones, 2009). In addition, it is believed that both SA and *t*JA are implicated in plant responses to abiotic stresses (Ahmad et al., 2016; Horvàth, Szalai, & Janda, 2007). The increase in SA and *t*JA indicated that CTV‐tALAD plants were under stress.

In agreement with our results, Chai et al. (2017) reported an increase in the level of ROS and H_2_O_2_ in *Gossypium hirsutum* (cotton) lesion mimic mutant (*Ghlmm*), which exhibited an accumulation of *δ*‐ALA as a result of low activity of the ALAD enzyme. An increase in methanedicarboxylic aldehyde (MDA) level was also observed in mutant leaves, indicating peroxidation of unsaturated membrane lipids in cells. Silencing of *ALAD* in *G. hirsutum* by the TRV‐based VIGS also resulted in the same phenotype observed in mutant plants and showed an accumulation of *δ*‐ALA and H_2_O_2_ (Chai et al., 2017). The mutant plant also accumulated SA and showed high expression levels of pathogenesis‐related genes (PR) and high resistance to *Verticillium dahliae* infection (Chai et al., 2017). In addition, modulation of ALAD activity was found to control programmed cell death (PCD) and PR expression (Chai et al., 2017). Our results, together with the previous results, showed that silencing of *ALAD* induced stress in plants.

The level of ABA decreased in leaves and apical meristems of CTV‐tALAD, and this decrease was consistent with the decrease in its precursor (violaxanthin). In agreement with the HPLC results, the gene expression results showed that ten genes implicated in the ABA‐biosynthetic pathway were downregulated in CTV‐tALAD plants. Previous studies showed that ABA deficiency reduces plant development and elongation (Nitsch et al., 2012). The decrease in ABA indicated that silencing of *ALAD* could affect plant development.

### 4.4 Silencing of *ALAD* altered the metabolite profile of citrus leaves

We performed a nontargeted metabolomic analysis on the volatile and nonvolatile profiles of CTV‐tALAD plants to gather more information about the effects of silencing of *ALAD* gene in plants. Our results showed that fumaric, succinic, and malic acids were increased in CTV‐tALAD plants. The increase in these metabolites could result from the increase in respiration rate, which can be increased to provide more energy during plant responses to stresses (Adi et al., 2012). Citric acid and fumaric acid also increased in *C*Las‐infected citrus plants (Albrecht, Fiehn, & Bowman, 2016). The accumulation of citric acid and fumaric acid was explained as an attempt to enhance nutrient uptake in response *C*Las‐infection (Albrecht et al., 2016).

The level of l‐asparagine increased in CTV‐tALAD plants compared with CTV‐wt plants. l‐Asparagine is synthesized in plants by amidation of aspartate using either ammonium or glutamine as ammonium donor (Gaufichon, Reisdorf‐Cren, Rothstein, Chardon, & Suzuki, 2010). l‐Asparagine is implicated in nitrogen transport, metabolism, recycling and its flow in response to biotic and abiotic stresses (Gaufichon et al., 2010). Light, nitrogen and carbon availability control the three‐asparagine synthetase genes in sunflower (*Helianthus annuus*), *HAS1*,* HAS1.1*, and *HAS2* (Herrera‐Rodríguez, Maldonado, & Pérez‐Vicente, 2004). Light and ample carbon supply induce *HAS2* to supply asparagine to support growth. On the other hand, darkness and a low carbon/nitrogen ratio activate *HAS1* and *HAS1.1* genes to convert excess nitrogen to l‐asparagine. Ammonium activates all three genes to enhance its detoxification. The increase in l‐asparagine level in CTV‐tALAD plants could result from the increase in ammonium, which occurs upon abiotic stresses (Herrera‐Rodríguez, Pérez‐Vicente, & Maldonado, 2007). It is also possible that the decrease in sucrose and starch decreased the carbon/nitrogen ratio and activated *HAS1* and *HAS1.1* genes to produce more l‐asparagine using glutamine as an ammonium donor. The increase in l‐asparagine is another indication that accumulation of *δ*‐ALA induces stress in plants.

### 4.5 Silencing of *ALAD* enhanced emission of leaf volatiles

Previous studies showed that plant volatiles are triggered by both biotic and abiotic stress (Arimura, Matsui, & Takabayashi, 2009). In agreement with the previous findings, our results showed that the emission of (*E*)‐*β*‐ocimene, (*E*)‐*α*‐bergamotene and (*E*)‐*β*‐farnesene was highly increased in the CTV‐tALAD plants compared to the CTV‐wt plants. The increased emission of these volatiles indicated that silencing of *ALAD* induced stress in CTV‐tALAD. (*E*)‐*β*‐farnesene and (*E*)‐*α*‐bergamotene emission were increased in *Pinus taeda* upon exposure to high light and temperature (Helmig, Ortega, Guenther, Herrick, & Geron, 2006). In addition, the emission of these two volatiles was also increased in *Z. mays* and *Nicotiana attenuata* upon wounding, herbivory, or application of methyl jasmonate (Gaquerel, Weinhold, & Baldwin, 2009). Terpenes can relieve abiotic stress either by enhancing membrane stability during transient heat stress or by quenching reactive oxygen species (Palmer‐Young et al., 2015). Accumulation of ROS upon silencing of *ALAD* (Chai et al., 2017) could be the direct cause of the observed increase in volatile emission (Vickers, Gershenzon, Lerdau, & Loreto, 2009).

## 5 CONCLUSION

**Figure 9:**
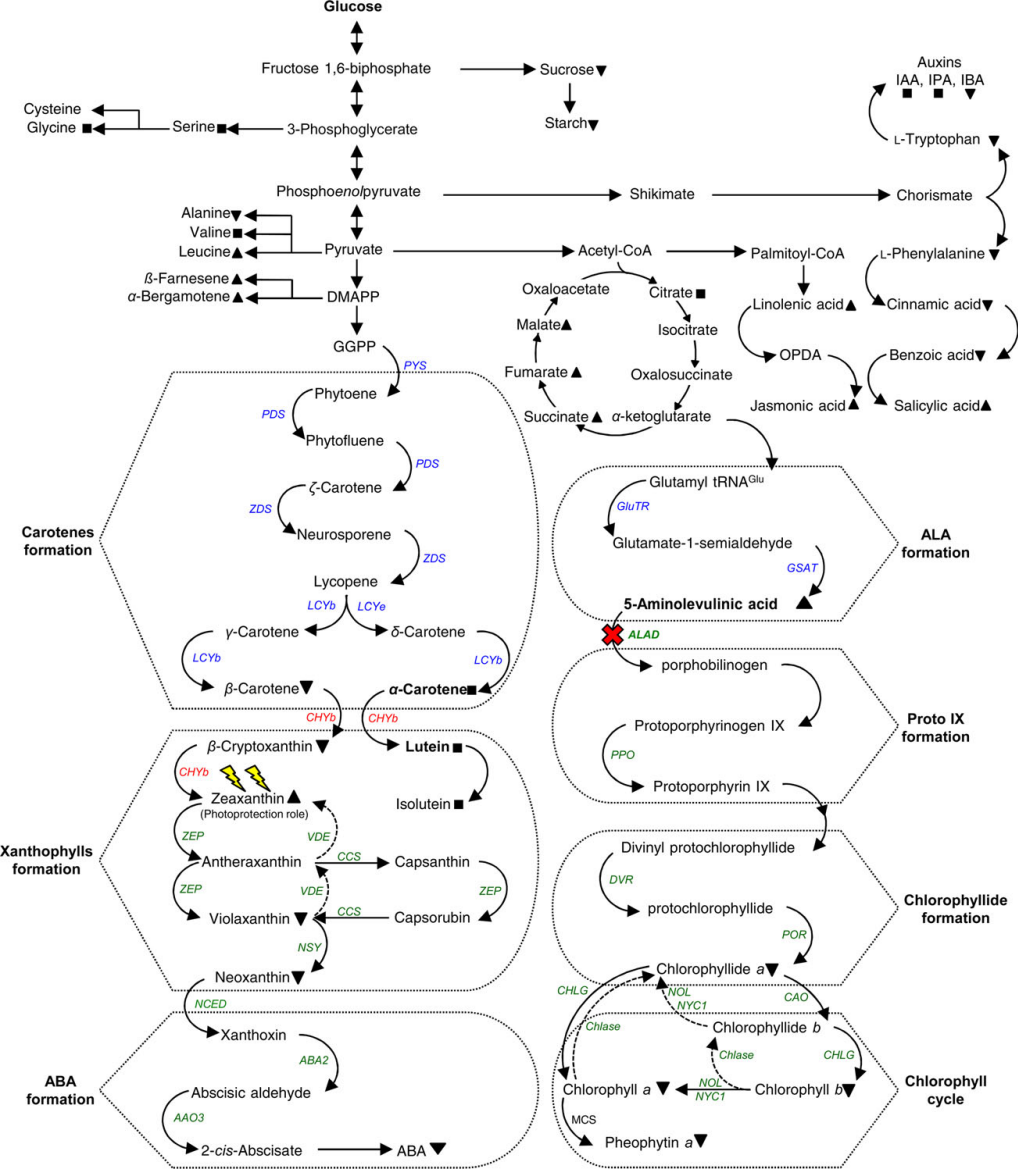
Schematic representation of proposed model for the effect of silencing of *ALAD* gene on *Citrus macrophylla* leaf pigments. Silencing of *ALAD* gene (red cross) results in accumulation of *δ*‐ALA and consequently reduces the rate of chlorophylls biosynthesis, photosynthates levels, and induces light stress in CTV‐tALAD plants. To prevent light damage, the CTV‐tALAD plant increases the rate of zeaxanthin accumulation. The increase in rate of zeaxanthin synthesis decreases the levels of its precursors, upstream pigments, and ABA. Silencing of *ALAD* gene also affected other phytohormones (BA, SA, *t*JA, IBA) and their precursors. In addition, silencing of *ALAD* gene affected the tricarboxylic acid cycle and enhanced the emission of several leaf volatiles. The decrease in upstream pigments reduces ABA synthesis. The up‐arrow (▲) indicates increasing, down‐arrow (▼) indicates decreasing, and square sign (■) indicates no changes in compound levels. The dotted lines with arrows represent hypothetical mechanisms or uncharacterized elements. Genes in red were upregulated, genes in green were downregulated, whereas those in blue were not affected. ABA: abscisic acid; DMAPP: dimethylallyl diphosphate; GGPP: trans‐geranyl‐geranyl diphosphate; IAA: indole‐3‐acetic acid; IBA: indole‐3‐butaric acid; IPA: indole‐3‐propionic acid; OPDA: 12‐oxo‐cis‐10,15‐phytodienoate

Silencing of *ALAD* in citrus plants using CTV‐tALAD vector resulted in accumulation of the toxic tetrapyrrole intermediate (*δ*‐ALA), decreased chlorophylls and photosynthate levels, altered the levels of carotenoids, phytohormones, volatiles, and many other metabolites and produced yellow islands and necrosis in citrus leaves. These effects are summarized in Figure 9. These changes showed that silencing of *ALAD* gene induces stress in plants.

## AUTHOR CONTRIBUTIONS

N.K. and S.G. planned and designed the research. N.K., F.H., Y.N., S.H., and S.G. performed experiments and analyzed data. N.K., F.H., Y.N., S.H., and S.G. wrote the manuscript.
