# Supplementary figures and images for: Effects of δ‐aminolevulinic acid dehydratase silencing on the primary and secondary metabolisms of citrus
Source: Plant Direct. 2018 Jul 16;2(7):e00072. doi: 10.1002/pld3.72 (PMC6508816; doi:10.1002/pld3.72)

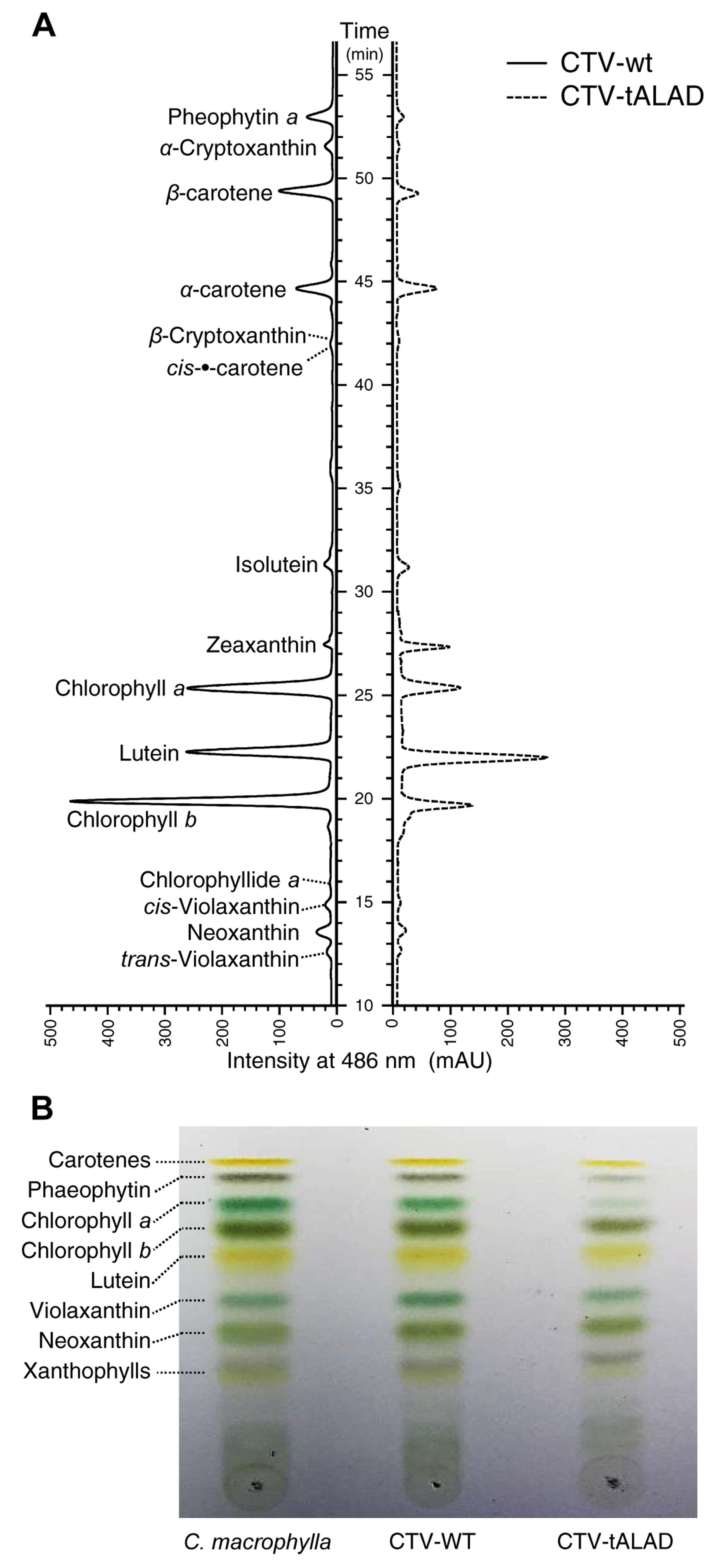

Supplement: Supplementary file 1 [file PLD3-2-e00072-s001.tif]

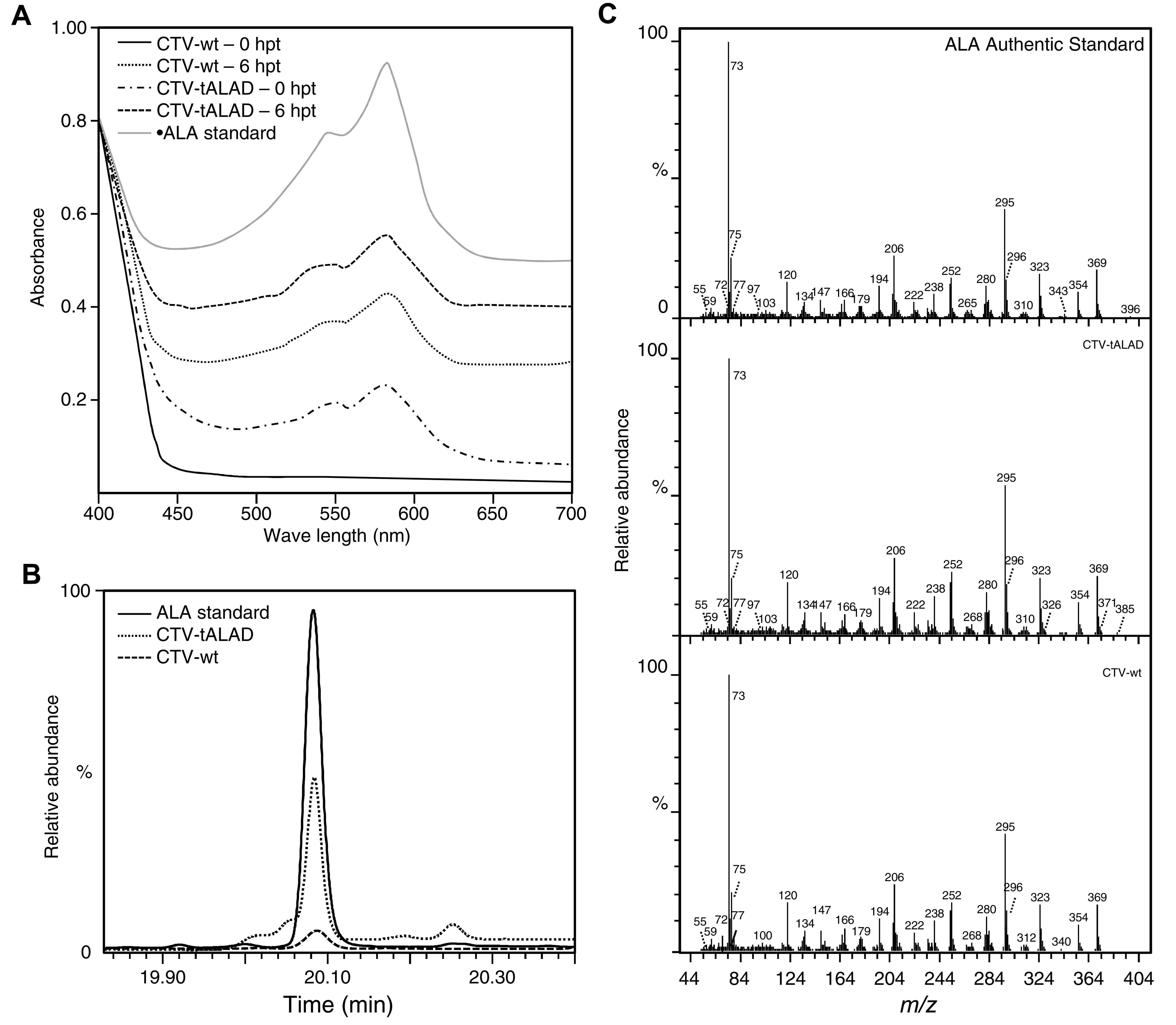

Supplement: Supplementary file 2 [file PLD3-2-e00072-s002.tif]
