# Supplementary material for: Effects of δ‐aminolevulinic acid dehydratase silencing on the primary and secondary metabolisms of citrus
Source: Plant Direct. 2018 Jul 16;2(7):e00072. doi: 10.1002/pld3.72 (PMC6508816; doi:10.1002/pld3.72)
Supplement: Supplementary file 3 [file PLD3-2-e00072-s003.pdf]

Dear Dr. Claudia Keitel

I cannot thank you enough for your great efforts in reviewing and editing our manuscript.

It was really great help. I anticipate that Plant direct will grow fast and it will be very successful journal in the field as long as great editors like you serve in the journal.

I did all your recommended corrections

All the best,

Nabil Killiny

-- Detailed editor comments:
Page 2
L9: discrete
L10: add "s" to level

Page 8
L7: leaf not leave

Page 10
L14 to L23: replace all instances of "conditions" with "concentrations"

Page 11
L3: capital letters for FW
L6: "FW" rather than "fwt"

Page 12
L2: All instances of L-(followed by an amino acid) anywhere in the manuscript should not be subscript.
L22 onwards; this section was substantially rephrased: "Consequently, we hypothesized that inhibition of this enzyme would not only have an impact on chlorophyll biosynthesis and photosynthesis, but also affect other pigments and could lead to changes in both primary and secondary metabolites. Therefore, we carried out targeted and non-targeted metabolic analysis on CTV-tALAD plants. The level of δ-ALA was determined to confirm the successful silencing of

the ALAD gene. We evaluated chlorophyll content since accumulation of δ-ALA was expected to reduce chlorophyll biosynthesis. In addition, we analyzed carotenoid content because carotenoids are functionally linked to chlorophyll in photosynthetic light harvesting. Reduction in chlorophyll biosynthesis was expected to decrease the rate of photosynthesis, thus we determined the level of the main photosynthates (sucrose and starch). CTV-tALAD plants showed clear symptoms of necrosis, which indicated they were under stress; therefore four different groups of phytohormones including the main growth regulators and stress-associated groups (auxins, salicylates, jasmonates, and ABA) were quantified. Finally, we performed non-targeted metabolomic analysis on volatile and non-volatile compounds in order to get more insights on the metabolic status of CTV-tALAD plants. Our results are discussed in the following paragraphs."

Page 14
L11: "silenced" (not silence)
L13: "plants than the inhibition" (plants not plant, add the)
L14 onwards: "The reduction in chlorophyll indicated that CTV-tALAD plants have a lower photosynthesis rate than control plants. In fact, our results showed that sucrose and starch levels (photosynthates) were reduced in CTV-tALAD plants. The reduction in chlorophylls reduces the amount of absorbed light and harvested energy (ATP and NADPH); consequently, carbon fixation is reduced and results in a decrease in photosynthates in leaves."
L22: This sentence too vague: "Carotenoids are closely linked with chlorophylls and work in coordination with these photosynthetic pigments." Please rephrase to "Interactions between chlorophylls and carotenoids play a major role in photosynthetic light harvesting"

Page 16
L14: Add "The" and "s" to response: "The SA pathway is associated with plant responses to biotrophic ..."
L16: Add "the" and "s" to response: "Contrarily, the tJA pathway is associated with plant responses to necrotrophic ..."
L18: plant responses

Page 17
L7: control (not controls)
L17: remove "also"
